# Differential lncRNA Expression in Undifferentiated and Differentiated LUHMES Cells Following Co-Exposure to Silver Nanoparticles and Nanoplastic

**DOI:** 10.3390/ma18122690

**Published:** 2025-06-07

**Authors:** Kamil Brzóska, Malwina Czerwińska, Marcin Kruszewski

**Affiliations:** 1 Centre for Radiobiology and Biological Dosimetry, Institute of Nuclear Chemistry and Technology, Dorodna 16, 03-195 Warsaw, Polandm.kruszewski@ichtj.waw.pl (M.K.); 2 Department of Molecular Biology and Translational Research, Institute of Rural Health, Jaczewskiego 2, 20-090 Lublin, Poland

**Keywords:** nanoplastic, silver nanoparticles, AgNPs, lncRNA, LUHMES cells

## Abstract

Human exposure to micro- and nanoplastic (MNP) has become an increasing concern due to its accumulation in the environment and human body. In the human organism, MNP accumulates in various tissues, including the central nervous system, where it is associated which neurotoxic effects. Beyond its inherent toxicity, MNP also acts as a carrier for various chemical contaminants, including metals. Consequently, recent studies emphasize the importance of the evaluation of co-exposure scenarios involving MNP and other types of nanoparticles. In this study, we investigated effects of co-exposure to 20 nm silver nanoparticles (AgNPs) and 20 nm polystyrene nanoparticles (PSNPs) on cell viability and the expression of inflammation-related long non-coding RNAs (lncRNAs) in undifferentiated and differentiated Lund human mesencephalic (LUHMES) cells. While PSNPs alone did not significantly affect cell viability or lncRNA expression, AgNPs markedly reduced viability and deregulated lncRNA expression in both cell types. Notably, in differentiated cells, co-exposure to AgNPs and high concentrations of PSNPs led to a significantly greater reduction in viability compared to AgNPs alone, suggesting a synergistic effect. At the molecular level, both synergistic and antagonistic interactions between AgNPs and PSNPs were observed in the regulation of lncRNA expression, depending on the cell differentiation status. These findings highlight the complex biological interactions between AgNPs and PSNPs and emphasize the importance of considering nanoparticle co-exposures in toxicological evaluations, as combined exposures may significantly affect cellular and molecular responses.

## 1. Introduction

The advancement of nanotechnology brings a range of innovations in medicine and industry, including the development of novel pharmaceuticals and advanced materials. However, alongside these benefits, concerns have emerged regarding the environmental implications of nanotechnology, particularly the increasing generation of nanowaste that consists of nanomaterials released into the environment either intentionally, unintentionally, or through the degradation of bulk materials [1]. A notable component of nanowaste is micro- and nanoplastic, (MNP) which is either deliberately produced for use in consumer products such as cosmetics, paints, personal care items, and textiles, or generated through the fragmentation of large plastic materials. The latter, referred to as secondary MNP, typically arises from the degradation of bulk plastics due to environmental factors such as ultraviolet (UV) radiation and mechanical abrasion. These particles pose a growing environmental concern due to their persistence, potential for bioaccumulation, and impact on ecosystems [2].

Humans are exposed to MNP through multiple pathways. Oral exposure is considered the primary route and may occur through the ingestion of contaminated food—particularly seafood—as well as drinking water, honey, sugar, and salt [3,4,5]. Inhalation exposure has also been documented, especially in indoor environments where MNP particles may be released from synthetic textiles or personal protective equipment, such as face masks [6]. Dermal exposure is possible through contact with contaminated water or the use of cosmetic products containing MNP; however, its relevance is considered limited due to the low permeability of intact human skin to micro- and nanoparticles [7]. MNP has been detected in various human organs and tissues, including lungs [8], blood [9], the placenta [10], testes, and semen [11,12]. Of particular concern is the potential accumulation of MNP in the central nervous system, including the brain. Postulated mechanisms of MNP neurotoxicity include the disruption of blood–brain barrier (BBB) integrity, induction of oxidative stress, activation of microglial cells leading to inflammation, and alterations in neurotransmitter levels. Additionally, MNP may disrupt gut microbiota, potentially affecting the gut–brain axis and contributing to neurological dysfunction [13,14,15]. Several studies have demonstrated the ability of MNP to cross the blood–brain barrier and accumulate in the brain of aquatic organisms, such as fish [16,17]. Similarly, in vivo studies on rats have confirmed the permeability of the blood–brain barrier to polystyrene nanoparticles [18]. Studies in mouse models have further demonstrated that polystyrene nanoparticles cross this barrier more efficiently than microparticles and that the composition of the biomolecular corona surrounding plastic particles plays a critical role in facilitating their passage through the blood–brain barrier [19]. Furthermore, the internalization of MNP by neuronal cells has been observed in vitro, indicating the potential for direct interactions with neural tissue [20,21].

Besides the potential hazard from the MNP itself, it can serve as a carrier for various chemical contaminants, including metals, persistent organic pollutants, and pathogenic microorganisms [22,23,24,25,26]. The adsorption of substances onto MNPs is highly efficient due to their large surface area. Adsorption may occur through either physical or chemical interactions, depending on the surface characteristics of the particles and the chemical properties of the substances being adsorbed [13]. Experimental studies increasingly emphasize the need to consider more complex exposure scenarios, such as co-exposure to MNP and other types of nanoparticles. Among the most widely produced and utilized nanoparticles are silver nanoparticles (AgNPs), and several studies have investigated the combined effects of MNP and AgNPs across different biological models. These studies frequently report significant interactions between the two particle types, suggesting potential synergistic or antagonistic effects that may influence toxicity outcomes [27,28,29,30,31].

In the present study, we contribute to this area of research by investigating the effect of co-exposure to AgNPs and polystyrene nanoparticles (PSNPs) on the expression of inflammation-related long non-coding RNAs (lncRNAs) in Lund human mesencephalic (LUHMES) cells. LUHMES cells are embryonic neuronal precursor cells that maintain constant proliferation in an undifferentiated state through the switchable expression of the v-MYC oncogene. Upon the addition of tetracycline, glial cell line-derived neurotrophic factor (GDNF), cAMP, and the removal of fibroblast growth factor (FDF) from the culture medium, these cells differentiate into morphologically and biochemically mature dopamine-like neurons. Differentiated LUHMES cells exhibit strong β-III-tubulin immunoreactivity, extensive neuritic processes, time-dependent induction of tyrosine hydroxylase, and extracellular dopamine release. Differentiation also leads to marked changes in the expression of several genes encoding ATP-binding cassette (ABC) transporters, which mediate the translocation of a wide range of compounds across cellular membranes, including inorganic anions, metal ions, sugars, peptides, and amino acids [32,33,34]. To account for potential differences in cellular responses based on the developmental stage, the effects of nanoparticle exposure were evaluated in both differentiated and undifferentiated LUHMES cells.

Briefly, lncRNAs are a class of transcripts exceeding 200 nucleotides in length and lacking conventional promoters, terminators, and open reading frames. Although they do not encode proteins, lncRNAs play crucial roles in the regulation of chromatin remodeling, DNA methylation, histone modification, and RNA metabolism. They are involved in key cellular processes such as cell survival, differentiation, and DNA repair [35,36]. These regulatory functions are mediated through interactions with proteins, DNA, other RNA molecules, or a combination of these components [37].

The involvement of lncRNAs in response to AgNPs has been demonstrated in several in vivo and in vitro models including mice [38], ciliates [39], rabbit fetal fibroblast cells [40], breast cancer cells [41], and K562 erythroid cells [42]. Similarly, alterations in lncRNA expression have been observed following exposure to nanoplastics in animal models, such as mice (in lung and brain tissues) [43,44] and in the nematode Caenorhabditis elegans [45].

To the best of our knowledge, the present study is the first to report changes in lncRNA expression in neuronal cells following AgNP and nanoplastic co-exposure, with a particular focus on potential interaction effects between the two nanoparticle types.

## 2. Materials and Methods

### 2.1. Nanoparticle Characterization

Citrate-coated AgNPs (20 nm nominal size) were purchased from NanoComposix (San Diego, CA, USA). Polystyrene nanoparticles (20 nm nominal size) were purchased from Nanocs (New York, NY, USA). The surface morphology of the samples was examined using a Carl Zeiss “ULTRA plus” high-resolution scanning electron microscope (Ultra-High-Resolution Imaging, Jena, Germany). Then, 100 µL of nanoparticles suspension was gently deposited onto aluminum specimen stubs (12.5 mm diameter, short pin). To ensure sample conductivity and minimize thermal damage during imaging, a thin carbon coating was applied using a JEE-4X vacuum evaporator (JEOL, Tokyo, Japan). Imaging was performed at an accelerating voltage of 3.00 kV and a working distance of 7.0 mm. The zeta potential of nanoparticles was determined by dynamic light scattering using the Zetasizer Nano ZS system (Malvern Instruments, Malvern, UK) at 25 °C in a DTS 1067 capillary cell. Working solutions of nanoparticles were diluted with deionized water. Each sample was measured in triplicate with 20 sub-runs to ensure reproducibility. Zeta potential values were calculated using the Smoluchowski approximation of the Henry equation (f(ka) = 1.5).

The hydrodynamic diameter of nanoparticles in cell culture media and PBS was analyzed using a NanoSight LM10 apparatus with Nanoparticle Tracking Analysis (NTA) 3.2 Build 16 software (Malvern Instruments, Malvern, UK). For cell culture treatment, both types of nanoparticles were vortexed for 30 s and diluted in the cell culture medium without additional processing.

### 2.2. Cell Culture

LUHMES cells were grown in a monolayer in cell culture flasks coated with poly-lornithine (50 μg/mL) and human plasma fibronectin (1 μg/mL) (Sigma Aldrich, St. Louis, MO, USA). Freshly coated flasks were used in each experiment. Undifferentiated, proliferating cells were grown in Advanced DMEM/F-12 medium (ThermoFisher Scientific, Waltham, MA, USA) supplemented with N-2 supplement (Sigma Aldrich, St. Louis, MO, USA), 40 ng/mL of basic fibroblast growth factor (bFGF) (ThermoFisher Scientific, Waltham, MA, USA), and 2 mM of l-glutamine (Sigma Aldrich, St. Louis, MO, USA). Differentiation was conducted according to the previously established two-step protocol [46]. Briefly, when cells reached 70% confluency the medium was changed to a differentiation medium containing 1 μg/mL of tetracycline, 1 mM of cAMP (Sigma Aldrich, St. Louis, MO, USA), and 2 ng/mL of GDNF (ThermoFisher Scientific, Waltham, MA, USA) instead of bFGF. On the next day (attributed as day 2 of differentiation), cells were trypsinized and seeded to the new flasks to create a monolayer of separated differentiated cells without visible clumps of unequally differentiated cells. Cells were differentiated for 7 days before performing the final experiment.

### 2.3. Neutral Red Assay

Undifferentiated or differentiated LUHMES cells were seeded in 96-well microplates (TPP, Trasadingen, Switzerland) at a density of 1 × 10^4^ cells/well or 5 × 10^4^ in 100 µL of culture medium, respectively. Twenty-four hours after seeding, cells were treated with nanoparticles. The following concentrations were used: 0.1–0.3 µg/mL of AgNPs and 12.5–100 µg/mL of PSNPs for undifferentiated cells; 3–9 µg/mL of AgNPs and 25–150 µg/mL of PSNPs for differentiated cells. After 24 h, the cell culture medium was removed, and the cells were incubated for 3 h at 37 °C with 100 µL of the neutral red (NR) solution at a final concentration of 40 μg/mL. Next, the NR solution was aspirated, cells were washed with 100 µL of PBS, and 150 µL of an acetic acid–ethanol solution (49% water, 50% ethanol, and 1% acetic acid) was added to each well. After 15 min of gentle shaking, fluorescence was read with excitation at 530 nm and emission at 645 nm in the Synergy H1 plate reader (Agilent BioTek, Santa Clara, CA, USA). Three independent experiments in three replicate wells were conducted.

### 2.4. Analysis of lncRNA Expression by Real-Time PCR

For gene expression experiments, cells were seeded on 6-well cell culture plates (8 × 10^5^ differentiated cells per well or 3 × 10^5^ undifferentiated cells per well). Subsequently, the fresh stock suspensions of nanoparticles were added to the cell cultures to obtain the following final concentrations: 4.2 µg/mL (0.932 µg/cm^2^, equal to 3 µg/mL on a 96-well plate) of AgNPs for differentiated cells; 0.28 µg/mL (0.062 µg/cm^2^, equal to 0.2 µg/mL on a 96-well plate) of AgNPs for undifferentiated cells; and 105 µg/mL (23.292 µg/cm^2^, equal to 75 µg/mL on a 96-well plate) of PSNPs for both cell types. After incubation for 6 h, the cells were harvested for RNA isolation. Total RNA was extracted from cell pellets using the ReliaPrep RNA Cell Miniprep System (Promega, Madison, WI, USA) according to the manufacturer’s protocol. RNA concentration was measured using a Quantus Fluorometer (Promega, Madison, WI, USA) and the QuantiFluor RNA System (Promega, Madison, WI, USA). One microgram of total RNA was reverse-transcribed and preamplified using the iScript Explore One-Step RT and PreAmp Kit (Bio-Rad, Hercules, CA, USA) following the manufacturer’s instructions. The cDNA was diluted ten times with deionized, RNAse-free H_2_O and used for lncRNA expression profiling using the Inflammation lncRNA H96 PrimePCR PCR Array (Bio-Rad, Hercules, CA, USA) according to the manufacturer’s instructions. Briefly, a total volume of 20 μL of PCR reaction mixture, which included 10 μL of SsoAdvanced Universal SYBR Green Supermix from BioRad, 8 μL of RNAse-free H_2_O, and 2 μL of diluted template cDNA was used for each primer set in each well of the PCR array. PCR amplification was carried out using the CFX96 Touch Real-Time PCR Detection System (Bio-Rad, Hercules, CA, USA) with an initial 2 min step at 95 °C followed by 40 cycles of 95 °C for 5 s and 60 °C for 30 s. Relative gene expression was calculated using the ΔΔCt method. B2M, HMBS, and TBP were used as reference controls. Calculations were performed using CFX Maestro 2.3 Software (Bio-Rad, Hercules, CA, USA).

### 2.5. Statistical Analysis

Statistical analysis was performed using R Statistical Software (version 4.4.3; R Foundation for Statistical Computing, Vienna, Austria) [47]. The following R packages were used: “ggplot2” version 3.5.1 [48], “ggVennDiagram” version 1.5.2 [49], and “pheatmap” version 1.0.12 [50]. Statistical significances were evaluated using *t*-test or ANOVA followed by Tukey’s post-hoc test. Differences were considered statistically significant when *p* < 0.05.

## 3. Results

### 3.1. Characterization of Nanoparticles

**Figure 1:**
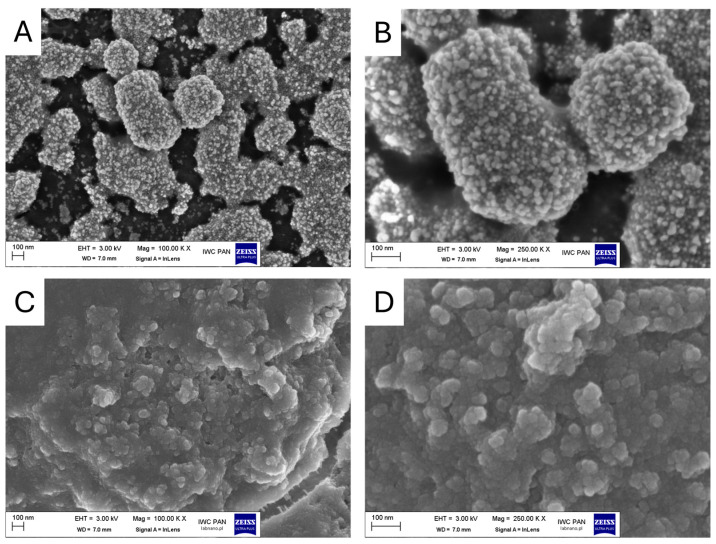
HR-SEM images of AgNPs (**A**,**B**) and PSNPs (**C**,**D**) used in the study.

Representative high-resolution scanning electron microscopy (HR-SEM) images of AgNPs are shown in Figure 1 at two magnifications: 100,000× (Figure 1A) and 250,000× (Figure 1B). The nanoparticles exhibit a predominantly spherical morphology with a tendency to form agglomerates. The surface of the aggregates appears rough and densely populated with smaller nanostructures, indicating a hierarchical structure of the agglomerates. Individual nanoparticles are discernible and are approximately 20 nm in diameter, consistent with the expected size range for AgNPs.

The HR-SEM images of the PSNPs reveal predominantly spherical shapes with smooth, well-defined surfaces. In the 100,000× magnification image (Figure 1C), the overall morphology and distribution of the nanoparticles can be observed. The surfaces of individual nanoparticles appear non-porous and compact, with no visible cracks or irregularities, indicating a high degree of structural integrity. In the 250,000× magnification image (Figure 1D), finer details of the nanoparticle morphology are evident. The spherical shape and smooth texture of the particles are confirmed, and the size distribution appears relatively narrow, with diameters ranging from approximately 50 nm to 100 nm. Some aggregation is present, likely due to sample drying during sample preparation.

The Zeta potential of AgNPs was −32.5 mV, as previously reported [51], while the Zeta potential of PSNPs was −40.02 mV. The hydrodynamic diameters of nanoparticles, measured by NTA in both cell culture medium and PBS, are presented in Table 1. As expected, hydrodynamic diameters of both types of nanoparticles were larger than their nominal sizes due to agglomeration and protein corona formation. To evaluate whether agglomeration is further enhanced when AgNPs and PSNPs are combined, the hydrodynamic diameter of the nanoparticle mixture was also measured. The mean hydrodynamic diameter of the AgNP–PSNP mixture was similar to that of AgNPs alone, indicating that combining both types of nanoparticles does not promote additional agglomeration.

### 3.2. Effect of AgNPs and PSNPs Treatment on Viability of Undifferentiated and Differentiated LUHMES Cells

**Figure 2:**
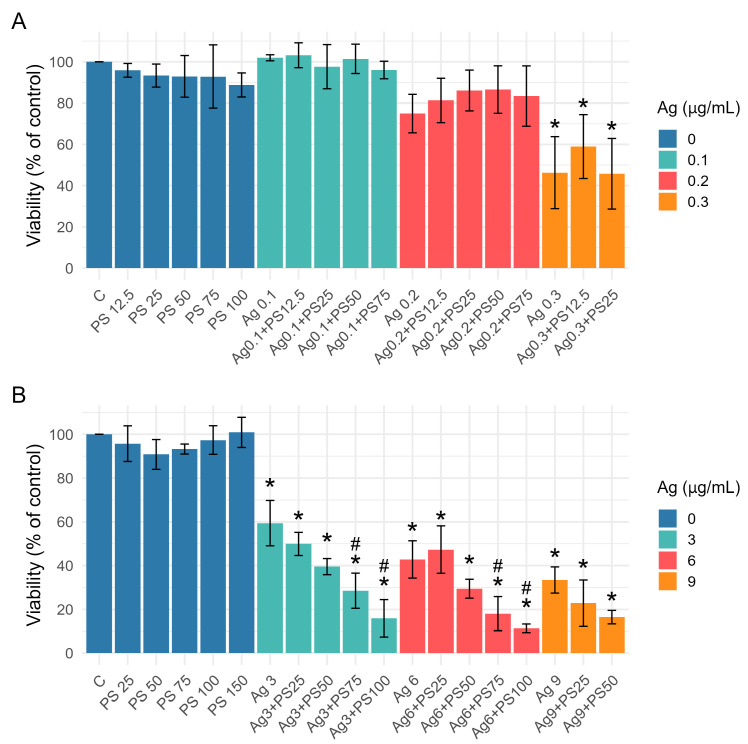
Viability of undifferentiated (**A**) and differentiated (**B**) LUHMES cells following exposure to AgNPs, PSNPs, or their combination for 24 h. Bars represent mean values, with error bars indicating the standard deviation from three independent experiments. Asterisks (*) denote statistically significant differences compared to control group (post-hoc Tukey test), while hash symbols (#) indicate statistically significant differences compared to the AgNP-treated group (post-hoc Tukey test).

Undifferentiated and differentiated LUHMES cells were exposed to various concentrations of AgNPs, PSNPs, and their mixture for 24 h. Subsequently, cell viability was assessed by means of a neutral red assay. Given the higher sensitivity of undifferentiated LUHMES cells to AgNPs, different concentration ranges were used for each cell type. The concentrations for differentiated cells were more than ten times higher than those for undifferentiated cells (3–9 µg/mL and 0.1–0.3 µg/mL, respectively). A two-way ANOVA indicated that AgNPs had a statistically significant effect on the viability of undifferentiated LUHMES cells, whereas PSNPs and the interaction between AgNPs and PSNPs did not show significant effects. Post-hoc Tukey’s test revealed that only the highest AgNP concentration, either alone or in combination with PSNPs, resulted in a significant reduction in viability compared to the control (Figure 2A).

In differentiated LUHMES cells, the effects of both AgNPs and PSNPs were statistically significant, as was their interaction. All tested concentrations of AgNPs led to a significant reduction in cell viability. While PSNPs alone did not affect viability, the combination of AgNPs with high PSNP concentrations resulted in a significantly greater decrease in viability compared to AgNPs alone, indicating a synergistic effect (Figure 2B).

Based on the cell viability analysis, the following nanoparticle concentrations were selected for lncRNA expression experiments: AgNP concentrations that resulted in a 30–40% reduction in cell viability (0.2 µg/mL for undifferentiated cells and 3 µg/mL for differentiated cells), as well as the lowest PSNP concentration for which a synergistic effect was observed (75 µg/mL). Because different culture vessels were used for lncRNA expression experiments, the concentrations were adjusted to maintain a consistent nanoparticle concentration per growth area across all experiments. The final concentrations used in the subsequent experiments were as follows: 4.2 µg/mL of AgNPs (0.932 µg/cm^2^, equivalent to 3 µg/mL in a 96-well plate) for differentiated cells; 0.28 µg/mL of AgNPs (0.062 µg/cm^2^, equivalent to 0.2 µg/mL in a 96-well plate) for undifferentiated cells; and 105 µg/mL of PSNPs (23.292 µg/cm^2^, equivalent to 75 µg/mL in a 96-well plate) for both cell types.

### 3.3. lncRNA Expression in Undifferentiated LUHMES Cells

**Figure 3:**
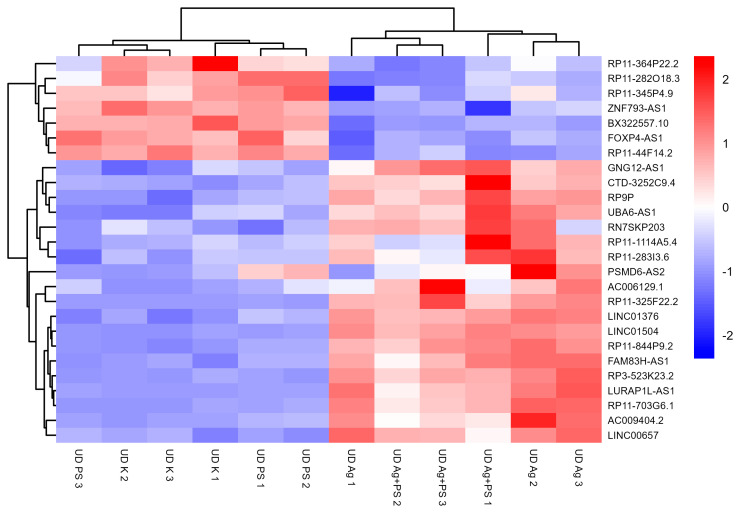
Heatmap of lncRNAs significantly deregulated in undifferentiated LUHMES cells after exposure to AgNPs or their mixture with PSNPs. Expression values are standardized per gene (row Z-score).

**Figure 4:**
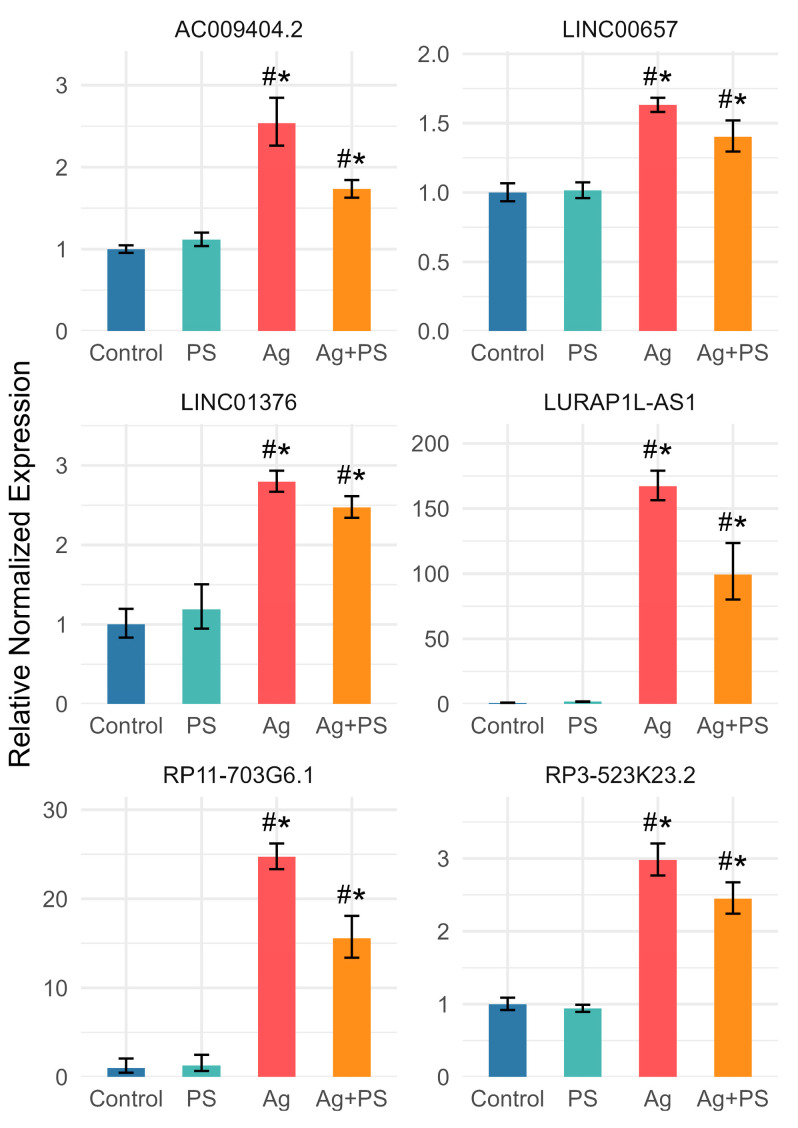
Expression of lncRNAs for which PSNPs suppressed AgNP-induced upregulation in undifferentiated LUHMES cells. Bars represent mean values, with error bars indicating the standard deviation from three independent experiments. Asterisks (*) denote statistically significant differences compared to control group (post-hoc Tukey test), while hash symbols (#) indicate statistically significant differences compared to the AgNP-treated group (post-hoc Tukey test).

A significant deregulation of lncRNA expression was observed in undifferentiated LUHMES cells following treatment with AgNPs or the combination of AgNPs and PSNPs, whereas no significant changes were detected in cells exposed to PSNPs alone. Fold changes and corresponding t-test *p*-values for all analyzed lncRNAs are provided in Supplementary Table S1. According to the *t*-test, 18 lncRNAs were deregulated relative to control following exposure to both AgNPs and the AgNP–PSNP mixture, while 3 lncRNAs were affected only by AgNPs, and 5 lncRNAs were altered exclusively in response to the AgNP–PSNP mixture. All lncRNAs significantly different from the control in at least one treatment group were used to generate a heatmap, presented in Figure 3. The heatmap reveals two main clusters of lncRNAs. The first cluster consists of lncRNAs downregulated by AgNPs and the AgNP–PSNP mixture. In contrast, the second cluster includes lncRNAs that were upregulated in response to AgNPs and/or their combination with PSNPs. Two-way ANOVA identified a significant interaction between AgNPs and PSNPs for six lncRNAs (LINC00657, LURAP1L-AS1, AC009404.2, RP3-523K23.2, LINC01376, RP11-703G6.1). In all cases, PSNPs suppressed AgNP-induced upregulation of these lncRNAs (Figure 4).

### 3.4. lncRNA Expression in Differentiated LUHMES Cells

**Figure 5:**
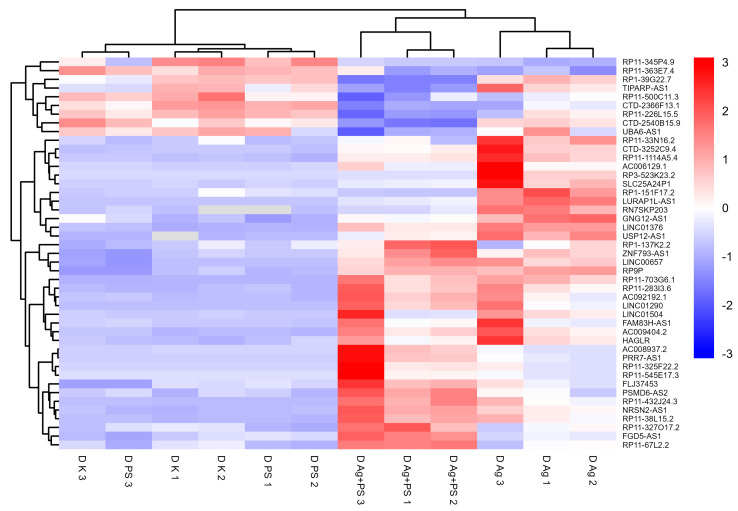
Heatmap of lncRNAs significantly deregulated in differentiated LUHMES cells after exposure to AgNPs or their mixture with PSNPs. Expression values are standardized per gene (row Z-score).

**Figure 6:**
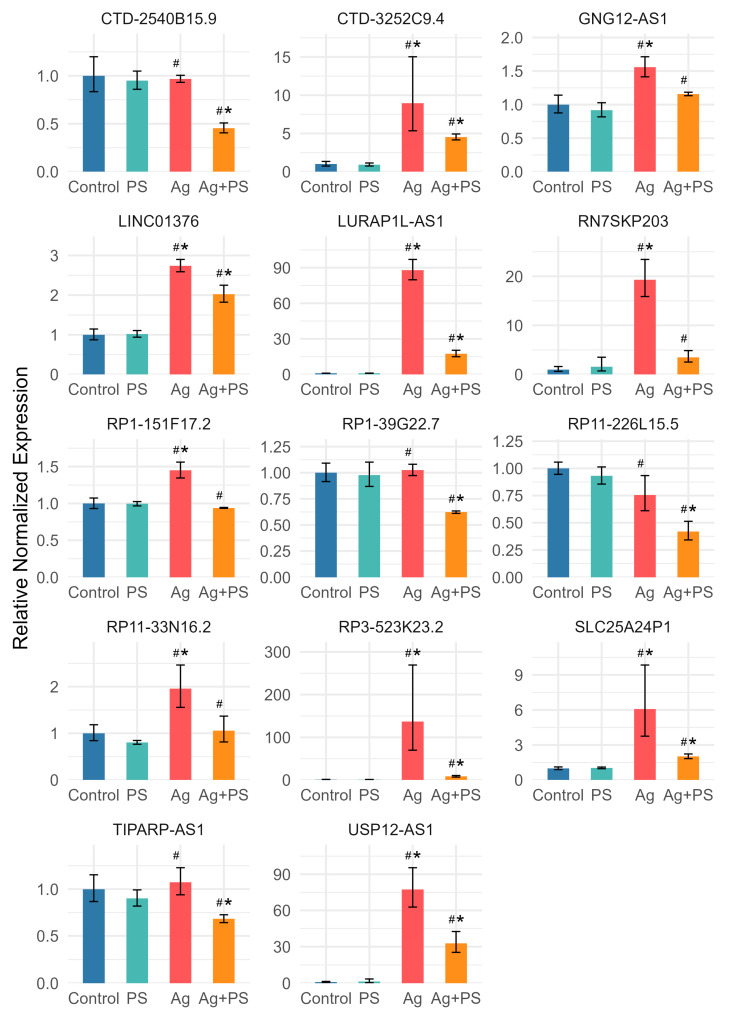
Expression of lncRNAs for which PSNPs suppressed AgNP-induced upregulation in differentiated LUHMES cells. Bars represent mean values, with error bars indicating the standard deviation from three independent experiments. Asterisks (*) denote statistically significant differences compared to control group (post-hoc Tukey test), while hash symbols (#) indicate statistically significant differences compared to the AgNP-treated group (post-hoc Tukey test).

**Figure 7:**
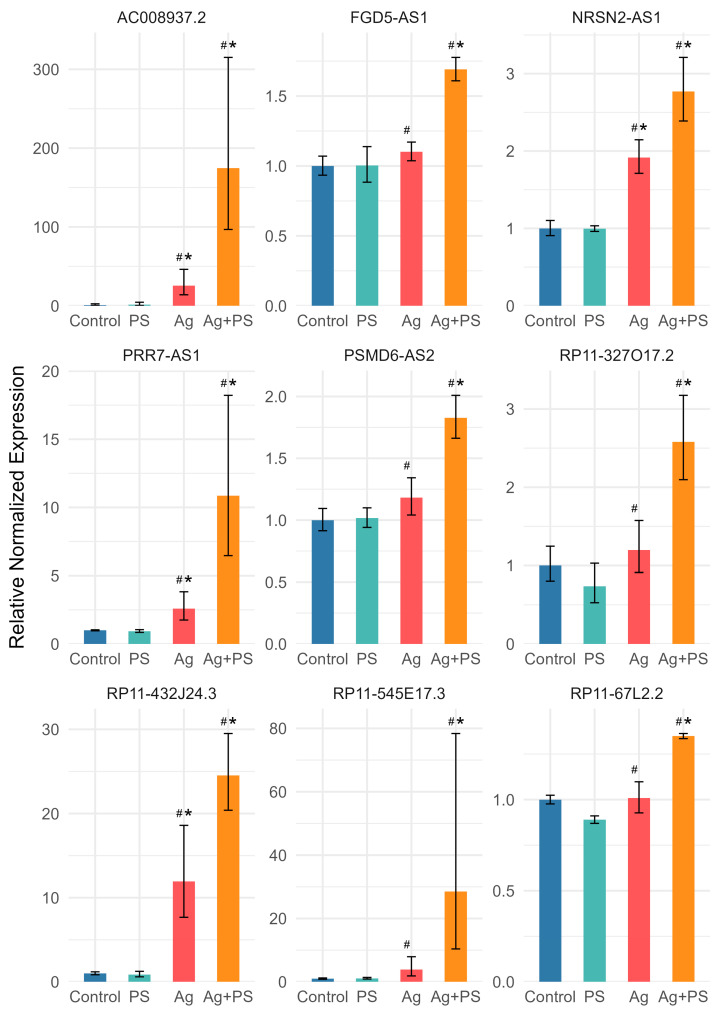
Expression of lncRNAs for which synergy of PSNPs and AgNPs was observed in differentiated LUHMES cells. Bars represent mean values, with error bars indicating the standard deviation from three independent experiments. Asterisks (*) denote statistically significant differences compared to control group (post-hoc Tukey test), while hash symbols (#) indicate statistically significant differences compared to the AgNP-treated group (post-hoc Tukey test).

Deregulation of lncRNA expression following exposure to AgNPs or their combination with PSNPs was more pronounced in differentiated LUHMES cells compared to undifferentiated cells. However, similar to undifferentiated cells, no significant changes were observed in cells exposed to PSNPs alone. Fold changes and corresponding t-test *p*-values for all analyzed lncRNAs are provided in Supplementary Table S2. According to the t-test, 24 lncRNAs were deregulated relative to the control following both AgNPs and the AgNP–PSNP mixture treatment, while 6 lncRNAs were affected only by AgNPs, and 16 lncRNAs were altered exclusively in response to the AgNP–PSNP mixture. All lncRNAs significantly different from the control in at least one treatment group were used to generate a heatmap, presented in Figure 5. The heatmap reveals four distinct clusters of lncRNAs. The first cluster consists of lncRNAs whose expression decreased following treatment with the AgNP–PSNP mixture, with either a similar or negligible effect observed for AgNPs alone. The remaining lncRNAs exhibited increased expression following exposure to AgNPs and/or the AgNP–PSNP mixture. Within this group, three subclusters were identified based on the effect of PSNPs: inhibition of AgNP-induced upregulation, a synergistic effect, or no effect. Two-way ANOVA identified a significant interaction between AgNPs and PSNPs for 23 lncRNAs. Among these, PSNPs suppressed AgNP-induced upregulation in 14 cases (Figure 6), while a synergistic effect of AgNPs and PSNPs was observed in 9 cases (Figure 7).

### 3.5. Comparison of lncRNA Expression in Undifferentiated and Differentiated LUHMES Cells

**Figure 8:**
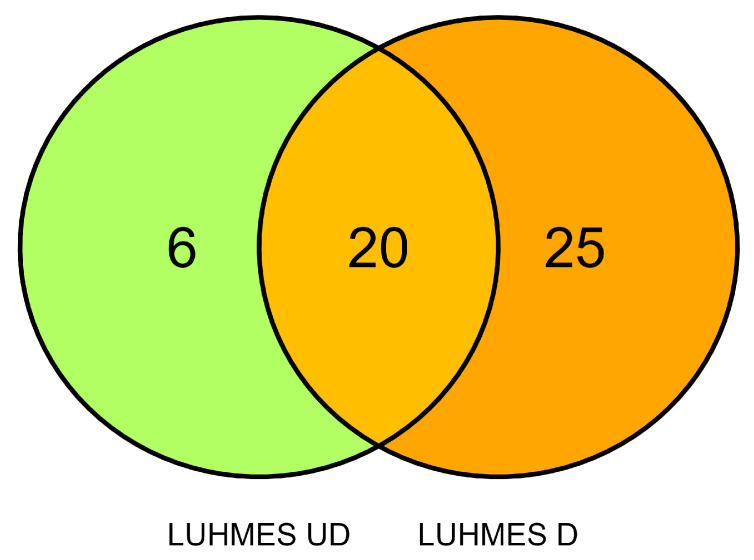
Venn diagram comparing lncRNA deregulation in undifferentiated (UD) and differentiated (D) LUHMES cells.

The Venn diagram in Figure 8 compares the effect of AgNPs and PSNPs on lncRNA expression in undifferentiated and differentiated LUHMES cells. Six lncRNAs were deregulated exclusively in undifferentiated cells, while 25 lncRNAs were deregulated only in differentiated cells. Twenty lncRNAs exhibited deregulation in both cel types, with 18 showing changes in the same direction, whereas UBA6-AS1 and ZNF793-AS1 displayed opposite patterns of deregulation (Supplementary Tables S1 and S2).

For three lncRNAs (LINC1376, LURAP1L-AS1, and RP3-523K23.2), significant interaction between AgNPs and PSNPs was observed in both undifferentiated and differentiated LUHMES cells. In all other cases, interactions were detected in only one of the two cell types.

## 4. Discussion

Nanoplastic and AgNPs are of particular concern from a human health perspective due to the increasing levels of both intentional and unintentional exposure. This growing exposure is largely driven by their high production volumes and widespread release into the environment. AgNPs are intensely manufactured and used primarily for their well-documented antibacterial properties, which have led to their incorporation into medical devices, textiles, and various consumer products. Unfortunately, the cytotoxic potential of AgNPs is not limited to bacteria, as they have also been shown to exert harmful effects on mammalian cells and tissues [52]. While the toxicological risks associated with AgNPs alone are well recognized and have been the subject of extensive research, less is known about the biological impact of co-exposure to AgNPs and other nanomaterials and nanoplastic in particular.

PSNPs are widely used as a model nanoplastic in toxicological research due to their availability in well-defined sizes and surface chemistries, which enable reproducible and controlled studies [13]. PSNPs also represent one of the major types of nanoplastic detected in the environment, as polystyrene is a commonly used polymer in industrial applications and consumer products. While nanoplastic in the environment may exhibit a broad range of chemical compositions and morphologies, studies using PSNPs provide a foundational understanding of the mechanisms of nanoplastic toxicity, which can inform and guide future research on more complex nanoplastic mixtures.

Domenech et al. demonstrated that AgNPs and PSNPs remain associated during their uptake by the Caco-2 cells. Although the internalization of AgNP–PSNP complexes did not result in increased cytotoxicity compared to the effects of each nanoparticle type alone, the complexes slightly altered the genotoxic potential of AgNPs [53]. Jurkat cells treated with the AgNP–PSNP mixture at concentrations that were not toxic when nanoparticles were applied alone showed reduced cell viability, increased reactive oxygen species (ROS) production, and apoptosis [28]. It has been reported that the presence of plastic particles aggravates the toxicity of AgNPs in mice, as evidenced by altered energy and choline metabolism following co-exposure to high doses of both materials [29]. Similar effects have been observed in ciliated protozoa and zebrafish, where nanoplastic enhanced toxicity and neurotoxicty induced by AgNPs [30,31]. Conversely, Alaraby et al. reported an antagonistic interaction between nanopolystyrene and AgNPs in Drosophila. In that study, co-exposure to polystyrene particles reduced the oxidative stress and genotoxicity associated with AgNP exposure. The authors attributed this antagonistic effect to the capacity of nanoplastic to sequester silver particles, thereby reducing their bioavailability and mitigating their toxic potential [27].

In the present study, undifferentiated and differentiated LUHMES cells exhibit markedly different responses to exposure to AgNPs and their mixture with PSNPs. While reduction in cell viability was observed in both cell types following AgNP exposure, undifferentiated cells demonstrated significantly higher sensitivity, with comparable effects occurring at concentrations approximately 15 times lower than those required to affect differentiated cells (Figure 2). Notably, a synergistic effect between AgNPs and PSNPs was observed only in differentiated cells, and only at higher concentrations of PSNPs. The higher sensitivity of undifferentiated LUHMES cells to AgNPs likely reflects fundamental differences in cellular physiology, metabolic activity, and stress response between the two cell states, as differentiation induces profound changes in gene expression, chromatin organization, and signaling pathways activity [54,55]. Undifferentiated LUHMES cells are actively proliferating, whereas differentiated cells are post-mitotic. Most mammalian neuron cells enter the post-mitotic state during the embryonic period. Since post-mitotic neurons are largely irreplaceable and must persist for the lifetime of the organism, they have evolved highly efficient defense mechanisms to support their sustained metabolic activity and ensure long-term survival [56]. It has been shown that differentiation of LUHMES cells is accompanied by significant upregulation of several members of the ABC family of proteins. ABC transporters play a critical role in cellular detoxification by actively effluxing a wide range of xenobiotics, including metal ions, out of the cell. The upregulation of these transporters in differentiated cells may contribute to reducing nanoparticle-induced cytotoxicity [34]. Actively dividing cells tend to be more vulnerable to cytotoxic stress, as they require precise coordination of DNA replication, cell cycle progression, and protein homeostasis. Therefore, AgNP-induced oxidative stress or DNA damage have a more pronounced effect on undifferentiated LUHMES cells, leading to increased cytotoxicity.

Differences in cellular physiology and metabolic activity may also contribute to the observation that the synergistic effect of AgNPs and PSNPs on reducing cell viability was evident only in differentiated cells (Figure 2). Alternatively, it is possible that such interaction effects occur only at higher levels of AgNP exposure—the levels that are already lethal to undifferentiated LUHMES cells. The synergistic effect likely arises from a combination of mechanisms, and their detailed deciphering requires further investigation. It can be hypothesized that PSNPs enhance the cellular uptake of AgNPs, leading to increased intracellular accumulation and bioavailability. Additionally, both nanoparticle types can induce oxidative stress, and their combined presence may result in a cumulative increase in ROS levels, overwhelming antioxidant defense. Furthermore, AgNPs and PSNPs may modulate different but overlapping cellular signaling pathways, amplifying pro-apoptotic and cytotoxic responses.

There is growing recognition that lncRNAs can serve as biomarkers of toxicological responses in various organisms, including humans. Dysregulation of lncRNAs expression has been implicated in a wide range of health outcomes, such as cancer, Alzheimer’s disease, cardiovascular disorders, and autoimmune conditions [57]. Moreover, altered lncRNA profiles have been reported following exposure to diverse environmental and chemical stressors, including polycyclic aromatic hydrocarbons, benzene, chlorpyrifos-methyl, bisphenol A, phthalates, phenols, cadmium, arsenic, mercury, particulate matter, AgNPs, and nanoplastic [38,39,40,41,42,43,44,45,57,58].

The regulatory complexity of lncRNAs adds to the challenge of elucidating their specific roles in toxicological responses. A single gene may be regulated by multiple lncRNAs, while one lncRNA can affect expression of numerous target genes. This intricate network of interactions complicates the identification and functional validation of lncRNA–target relationships and presents a barrier to establishing lncRNAs as early, specific biomarkers for toxicity and disease development. Nonetheless, exploratory studies, such as ours—focusing on lncRNA expression following co-exposure to multiple nanoparticles—are essential to build the foundational data required for future risk assessment frameworks and regulatory decision making.

The main purpose of this work was to check whether the response of neural cells to silver and plastic nanoparticles is accompanied with deregulation of lncRNA expression. Our results confirmed that response to AgNPs alone or co-exposed with PSNPs involves significant deregulation of lncRNA expression. Notably, no changes were observed after exposure to PSNPs alone, which is consistent with cell viability data showing no significant effect of PSNPs. This contrasts with previous studies reporting neurotoxic effects of PSNPs. For example, Jeong et al. demonstrated that developmental exposure to PSNPs in an in vivo mouse model led to abnormal neurodevelopment, ultimately causing brain dysfunction and cognitive impairment. Similarly, PSNP-induced molecular and functional alterations have been reported in cultured neural stem cells in the same study [59]. Shan et al. also observed PSNP accumulation in the brains of exposed mice, accompanied by microglial activation and neuronal damage. Furthermore, in vitro studies using immortalized human cerebral microvascular endothelial cells revealed that PSNP exposure can induce ROS production, activate the NF-κB pathway, and trigger necroptosis [60]. Both studies used PSNP concentrations similar to those applied in our study; it seems, therefore, that the biological effects of PSNPs are highly context-dependent, varying according to the experimental model and specific physicochemical characteristics of the nanoparticles used.

In both undifferentiated and differentiated LUHMES cells, several clusters of lncRNAs were identified, each exhibiting distinct expression patterns. As could be expected from analysis of viability, deregulation of lncRNA expression was more pronounced in differentiated LUHMES cells. The interpretation of the results is hampered by the fact that the functions of most lncRNAs have not yet been elucidated. Nevertheless, the function of several lncRNAs is known quite well, of which of particular interest in the context of the present study are LURAP1L-AS1 and LINC00657.

LURAP1L-AS1 targets the Leucine-rich adaptor protein 1-like (LURAP1L) gene, a known activator of the canonical NF-κB signaling pathway [61]. Overexpression of LURAP1L-AS1 leads to increased expression of LURAP1L, thereby enhancing NF-κB pathway activation [62]. In the present study, exposure to AgNPs resulted in strong upregulation of LURAP1L-AS1 in both undifferentiated and differentiated LUHMES cells. This observation aligns with previous reports demonstrating NF-κB activation following AgNP exposure in various biological models [63,64,65]. Interestingly, co-exposure with polystyrene nanoparticles (PSNPs) significantly suppressed the AgNP-induced upregulation of LURAP1L-AS1 in both cell types, suggesting a potential reduction in NF-κB pathway activation under combined exposure conditions. Given that canonical NF-κB signaling is generally associated with pro-survival, pro-proliferative, and anti-apoptotic effects, it is plausible to hypothesize that the observed inhibition of LURAP1L-AS1 expression—and consequently reduced NF-κB activation—may contribute to the decreased cell viability seen following co-exposure to AgNPs and PSNPs. This effect was particularly pronounced in differentiated LUHMES cells, which may reflect cell-type-specific sensitivity to modulation of survival pathways.

LINC00657, also known as “non-coding RNA activated by DNA damage” (NORAD), is a p53-dependent lncRNA that is broadly and abundantly expressed across mammalian cells and tissues. NORAD plays a critical role in maintaining genomic stability by serving as a multivalent binding platform for PUMILIO family RNA-binding proteins [66]. Interestingly, it was reported that LINC00657 is induced by hypoxia or oxidative stress and is implicated in regulation of apoptosis and inflammation via the NF-κB signaling pathway [67,68,69], suggesting broader involvement of LINC00657 in cellular stress responses. Our findings support these roles, as we observed increased expression of LINC00657 in both undifferentiated and differentiated LUHMES cells following exposure to AgNPs and the AgNP–PSNP mixture (Supplementary Tables S1 and S2). The increase was slightly more pronounced in differentiated cells. However, in undifferentiated cells, co-exposure with PSNPs significantly attenuated the AgNP-induced upregulation of LINC00657. While this interaction effect was statistically significant, it was relatively modest, and its biological relevance remains uncertain and warrants further investigation (Figure 4).

Taken together, our findings reveal complex interactions between silver and plastic nanoparticles in regulating lncRNA expression. While some lncRNAs show synergistic upregulation under combined exposure, others exhibit a suppressed response, suggesting that PSNPs can both enhance and mitigate AgNP-induced gene regulation. It is likely due to the complexity of lncRNA regulatory networks. Each lncRNA is controlled by a distinct combination of transcription factors, epigenetic modifications, and chromatin architecture; therefore, the interplay between PSNPs and AgNPs does not affect all lncRNAs uniformly. These results underline the need to consider nanoparticle co-exposure effects in toxicological assessments, as interactions between different types of nanoparticles may significantly alter cellular responses. Future research should focus on elucidating the precise molecular mechanisms underlying these interactions and their potential implications for environmental and human health. The heightened sensitivity of undifferentiated LUHMES cells to AgNP exposure underscores the importance of cellular state in nanoparticle toxicity. Understanding these differences is crucial for assessing nanomaterial safety, particularly in the context of neurodevelopmental toxicity and regenerative medicine applications.

## Figures and Tables

**Table 1:** Hydrodynamic diameter of PSNPs, AgNPs, and their mixture in PBS and Advanced DMEM/F-12 medium. Means ± standard deviation from three independent experiments.

Medium	Parameter	PSNPs	AgNPs	PSNPs + AgNPs
PBS	Mean (nm)	159 ± 18	251 ± 47	233 ± 18
	Mode (nm)	120 ± 21	182 ± 14	170 ± 11
DMEM/F-12	Mean (nm)	92 ± 1	280 ± 32	289 ± 50
	Mode (nm)	87 ± 7	233 ± 57	281 ± 43

## Data Availability

The original contributions presented in this study are included in the article/Supplementary Materials. Further inquiries can be directed to the corresponding author.

## Supplementary Materials

The following supporting information can be downloaded at: https://www.mdpi.com/article/10.3390/ma18122690/s1, Table S1: lncRNA expression in undifferentiated LUHMES cells following exposure to AgNPs, PSNPs or the mixture of both; Table S2: lncRNA expression in differentiated LUHMES cells following exposure to AgNPs, PSNPs or the mixture of both; Table S3: Viability of undifferentiated and differentiated LUHMES cells measured by Neutral Red assay following exposure to AgNPs, PSNPs or their combination for 24 h.
